# Therapeutic Outcomes of Oral Antibiotics for the Treatment of Methicillin-Resistant *Staphylococcus aureus* Urinary Tract Infections: A Multisite, Retrospective Cohort Study of Linezolid, Trimethoprim–Sulfamethoxazole and Doxycycline

**DOI:** 10.3390/antibiotics15100956

**Published:** 2026-09-25

**Authors:** Ryan McCall, Elena Gatskevich, Steven E. Fiester, Michael Floren, Elisabeth Chandler

**Affiliations:** 1 Department of Pharmacy, Lee Health, 13681 Doctors Way, Fort Myers, FL 33912, USA; elisabeth.chandler@leehealth.org; 2 Lee Physician Group, Infectious Diseases, Lee Health, 13681 Doctors Way, Fort Myers, FL 33912, USA; 3 Department of Biological Sciences, Florida Gulf Coast University, Fort Myers, FL 33965, USA; 4 Family Medicine Residency Program at Lee Health, College of Medicine, Florida State University, Fort Myers, FL 33901, USA; 5 Academic Medicine Enterprise, Lee Health, Fort Myers, FL 33912, USA; 6 Department of Pathology, Prisma Health Upstate, Greenville, SC 29605, USA; 7 Internal Medicine Residency Program at Lee Health, College of Medicine, Florida State University, Cape Coral, FL 33990, USA; 8 Department of Chemistry, Furman University, Greenville, SC 29613, USA; 9 School of Health Research, Clemson University, Clemson, SC 29634, USA; 10 Department of Finance, Economics, and Data Analytics, University of North Alabama, Florence, AL 35632, USA

**Keywords:** methicillin-resistant *Staphylococcus aureus*, oral antibiotic, urinary tract infection, linezolid, trimethoprim–sulfamethoxazole, doxycycline

## Abstract

Background: Methicillin-resistant *Staphylococcus aureus* (MRSA) urinary tract infections (UTIs), while relatively uncommon, pose a unique challenge in treatment as only specific intravenous (IV) options are typically utilized, with the efficacy of oral antimicrobials being poorly defined. Methods: The multi-site retrospective cohort study described herein therefore aims to characterize outcomes associated with oral administration of linezolid, trimethoprim-sulfamethoxazole (TMP-SMX), or doxycycline for the treatment of MRSA UTIs. Study participants were adults with culture-positive MRSA UTIs receiving one of the three oral therapies. Exclusion criteria included polymicrobial UTI, concomitant MRSA outside the urinary tract, receipt of non-study anti-MRSA agents for greater than or equal to 50% of treatment, less than 48 h of the oral study drug, and/or prostatitis. The primary outcome was clinical cure defined as a composite of symptom resolution, no readmission within 30 days of discharge attributable to UTI, and no change in therapy due to treatment failure. Secondary outcomes included antibiotic discontinuation because of adverse effects (AEs). Results: Overall, patients attained a clinical cure rate from MRSA UTI of 78.8%, 80.0%, and 94.4% when treated with linezolid, TMP-SMX, or doxycycline, respectively. Conclusions: While there were no observed differences between the clinical cure rates of the three antimicrobials studied, the high clinical cure rate coupled with low rates of AEs may support these oral therapies for the treatment of MRSA UTIs, but further investigation is required.

## 1. Introduction

Methicillin-resistant *Staphylococcus aureus* (MRSA) is an uncommon but clinically relevant cause of urinary tract infections (UTIs), with epidemiologic studies estimating an incidence of approximately 0.8 to 2% of all urinary cultures [1,2]. Innate risk factors for developing an MRSA UTI include exposure to healthcare institutions, prior isolation of MRSA in the urine, and MRSA bacteremia. Independent risks associated with MRSA UTIs involve indwelling catheterization, kidney stones (nephrolithiasis), and kidney swelling due to an inability of urine to drain from the kidney(s) to the bladder (hydronephrosis) [3]. MRSA in the urine is clinically significant as it can be a marker of more systemic MRSA infection [4,5]. Patients with positive MRSA urine cultures often have infectious disease specialty consultation and longer lengths of stay [6,7].

MRSA poses a unique challenge when present in the urine, as outside of intravenous (IV) options, there are limited efficacy data on oral treatment options. Increasing resistance has led to fluoroquinolones no longer being adequate empiric options. Both resistance and significant urinary excretion concerns limit clindamycin’s utility in this setting as well. The use of nitrofurantoin is limited to uncomplicated UTIs (infections of the lower urinary tract) due to the lack of therapeutic concentrations outside of the bladder. This has led to oral therapeutic options becoming limited to trimethoprim-sulfamethoxazole (TMP-SMX), doxycycline, and linezolid [8]. The majority of clinical data support the use of TMP-SMX, a sulfonamide derivative that interferes with bacterial folic acid synthesis, resulting in bactericidal activity. TMP-SMX has shown both in vitro and in vivo efficacy against MRSA isolates and infections [9,10]. Urine recovery of TMP-SMX is 84.5% for total sulfonamide and 66.8% for free trimethoprim after one dose [11]. There are significant patient-specific concerns associated with the use of TMP-SMX, however, such as hypersensitivity reactions, acute kidney injury, and metabolic abnormalities. Risks associated with TMP-SMX have led to increased use of alternative agents.

Doxycycline has been proposed as one potential alternative, but increasing resistance to common uropathogens and limited urinary excretion have led to its use falling out of favor for the treatment of UTIs [9,12]. Doxycycline is a tetracycline antibiotic that binds bacterial ribosomal subunits 30S and possibly 50S, leading to its bacteriostatic activity. Doxycycline has a reported urinary excretion of roughly 30%, which may lead to subtherapeutic concentrations depending on the reported organism’s minimum inhibitory concentration (MIC) [12].

Another alternative, linezolid, is an oxazolidinone antibiotic that halts bacterial growth through binding of the 23S ribosomal RNA of the 50S subunit of bacterial ribosomes and is considered bacteriostatic against enterococci and staphylococci, but bactericidal against streptococci. Linezolid has not been historically recommended for therapeutic use for UTIs, as its predicted urinary excretion is 30–35% [13,14,15]. Recent retrospective and observational studies suggest that linezolid demonstrates comparable clinical outcomes to alternative agents for the treatment of vancomycin-resistant *Enterococcus* (VRE) UTIs, with similar clinical cure rates, low rates of antibiotic reinitiation, and infrequent clinical failure, supporting its potential efficacy despite limited prospective data [16,17,18]. Despite the scant clinical data supporting their use for MRSA UTIs, doxycycline and linezolid are often used in practice due to the need for oral options in these patients. This retrospective cohort study, from 1 January 2022 to 30 October 2025, therefore compares clinical success rates among MRSA UTIs treated with linezolid, doxycycline, or TMP-SMX as definitive oral therapy.

## 2. Results

In the study timeframe, 444 patients were identified, all of whom met inclusion criteria. Three hundred and fifty-three patients were excluded based on the criteria outlined in the Section 4. Of the 91 patients included, 57 were in the linezolid group, 26 in the doxycycline group, and eight in the TMP-SMX group, respectively (Figure 1 and Figure 2). Of the 91 included patients, the mean age was 79 years; 69.2% were male, 62.6% had a history of recurrent UTIs, and 49.4% were diagnosed with a complicated UTI (primarily due to the presence of fever or pyelonephritis), with no significant differences between the three treatment groups. Patients commonly exhibited healthcare-associated risk factors such as recent hospitalization within 90 days of admission (70.3%), recent intravenous antibiotic use within 90 days of admission (62.6%), catheterization prior to admission (18.6%), and prior isolation of MRSA in the urine (26.4%). Approximately 80.2% of patients had baseline urinary symptoms, such as dysuria, fever, increased frequency, or costovertebral tenderness. The remaining 19.8% of asymptomatic bacteriuria (ASB) patients, while not included in the primary analysis (unless they failed another component of the primary outcome), were included for secondary outcome analysis to allow for a more real-world patient population. These patients do not, however, represent a true urinary tract infection based on guideline definitions. It was common for the ASB patients to have diagnosed altered mental status (AMS) on admission, but due to historical evidence and guidelines, this was not included in this study as a criterion for symptomatic UTI.

Patients in the linezolid group were less likely to have ASB (10.5%) compared to the TMP-SMX (37.5%) and doxycycline (34.6%) groups (*p* ≤ 0.05). Post-hoc analysis demonstrated differences in ASB between linezolid and TMP-SMX (*p* ≤ 0.05) and linezolid and doxycycline (*p* ≤ 0.05), but not between doxycycline and TMP-SMX (*p* = 0.88). Duration of anti-MRSA therapy was numerically shorter in the TMP-SMX group (6 days) compared to the linezolid (10 days) or doxycycline (9.4 days) groups (*p* ≤ 0.05). Post-hoc pairwise comparisons demonstrated a difference in duration between TMP-SMX and linezolid (*p* ≤ 0.05), but not between TMP-SMX and doxycycline (*p* = 0.29) or linezolid and doxycycline (*p* = 0.76). Recurrent UTI, defined as two or more UTIs in six months or three or more UTIs in 12 months, was common amongst the study population, with 63.2%, 87.5%, and 53.9% of patients in the linezolid, TMP-SMX, and doxycycline groups, respectively, meeting this criterion. There were no observed differences between the groups regarding chronic comorbidities, including chronic kidney disease, hypertension, diabetes mellitus, or chronic catheterization. Empiric therapy against MRSA was started in 60.4% of patients, with no observed differences between groups. Patients were often on initial IV therapy 70/91 (76.9%) (*p* = 0.59), and agents most typically used were vancomycin in 24/70 patients (34.3%), linezolid in 18/70 (25.7%) patients, daptomycin in 6/70 (8.6%), or doxycycline in 3/70 (4.2%) patients, leaving 19/70 (27.1%) patients on initial IV therapy not covering MRSA. Doxycycline was dosed as 100 mg twice daily, linezolid was dosed as 600 mg twice daily, and TMP-SMX was dosed appropriately based on renal dysfunction in all 8 patients, often at 1 double-strength tablet twice daily. Duration of therapy was calculated based on the addition of all IV and oral therapy active against MRSA, including discharge therapy. No differences were observed for the remaining baseline characteristics, as seen in Table 1.

Of the 75 patients included for the primary outcome, 78.8% of linezolid (41/52, 95% CI 66.0–87.8%), 80% of TMP-SMX (4/5, 95% CI 37.6–96.4%), and 94.4% of doxycycline (17/18, 95% CI 74.2–99.0%) treated patients attained clinical cure (*p* = 0.24) as shown in Table 2. Patients were readmitted for reasons attributable to UTI in 15.8% of linezolid patients (9/57, 95% CI 8.5–27.4%), 0% of TMP-SMX patients (0/8, 95% CI 0.0–32.4%), and 0% of doxycycline (0/26, 95% CI 0.0–12.9%) patients (*p* = 0.07). For symptomatic patients, symptoms resolved in 94.1% of linezolid patients (48/51, 95% CI 84.1–98.0%), 80% of TMP-SMX patients (4/5, 95% CI 37.6–96.4%), and 94.1% of doxycycline (16/17, 95% CI 73.0–99.0%) patients (*p* = 0.37). Therapy was discontinued in 1.9% of linezolid patients (1/57, 95% CI 0.3–9.3%), 0% of TMP-SMX patients (0/8, 95% CI 0.0–32.4%), and 3.8% of doxycycline (1/26, 95% CI 0.7–18.9%) patients due to lack of improvement (*p* = 0.61). AEs that led to discontinuation occurred in 3.5% of linezolid patients (2/57, 95% CI 1.0–11.9%), and 0% of TMP-SMX (0/8, 95% CI 0.0–32.4%) and 0% of doxycycline (0/26, 95% CI 0.0–12.9%) patients (*p* = 1.00).

Thrombocytopenia was reported in 4/57 (7.0%) patients on linezolid, gastrointestinal upset occurred in 1/26 (3.8%) doxycycline patients, and *Clostridioides difficile* infection occurred in 1/8 (12.5%) TMP-SMX patients and 1/26 (3.8%) doxycycline patients. Since linezolid susceptibility testing was not routinely reported for urinary isolates, available microbiologic data were limited. Of the available susceptibilities reported for TMP-SMX and doxycycline, 17/91 isolates were resistant to tetracycline (18.7%), and 29/91 (31.8%) were resistant to TMP-SMX. Tetracycline susceptibility was used to infer susceptibility to doxycycline per the Clinical & Laboratory Standards Institute [19]. Three patients in the doxycycline group were on resistant empiric therapy. No patients were on definitive resistant therapy in the doxycycline or TMP-SMX groups, but this is unknown for the linezolid group.

## 3. Discussion

While no observed differences in clinical cure rates were seen among patients in this retrospective cohort study evaluating oral antimicrobial therapy for MRSA UTIs between treatment groups, the clinical cure rate for the three studied antimicrobials ranged from 78.9% to 80% for linezolid and TMP-SMX, respectively, to 94.4% for doxycycline. The interpretation of these cure rates must be contextualized within the limitations of this retrospective, non-randomized trial as discussed below. Reported clinical cure rates for MRSA UTIs with intravenous treatment options remain limited, with a small prospective study of Gram-positive complicated UTIs (including MRSA) reporting a cure rate of roughly 85% with intravenous vancomycin therapy [20]. However, the cure rate should not be directly compared to the cure rate in this study, as no IV comparator group was included. Thus, this study provides insight into the potential value and efficacy of oral antimicrobial therapy. There were similarly no observed differences in secondary outcomes including UTI-related readmission, symptom resolution, therapy modification due to failure, or discontinuation due to adverse events between the three antimicrobial treatment groups. 

One important distinction between the three groups is the duration of therapy. Duration of therapy differed between the groups, specifically with TMP-SMX being associated with a median of 6 days, compared to numerically longer durations in the doxycycline and linezolid groups, 9.4 and 10 days, respectively. This difference in duration is likely a product of the small sample size; however, it may also reflect the desire to use a short course of TMP-SMX in an elderly population due to potential adverse effects. Durations of therapy overall in the study reflect commonly used durations in clinical practice, varying from 7–14 days. The selection of duration of therapy is often heavily reliant on patient-specific factors, clinical improvement, and provider experience; thus, interpretation of the impact of duration of therapy in terms of this trial is limited due to its retrospective nature. The exclusion of patients who received less than 48 h of therapy must also be addressed. This exclusion was most often applied to patients for whom it was decided that treatment was not indicated shortly after initiation, and to those with short durations of therapy due to low severity of illness. It was thus decided that excluding these patients would remove potential confounders. It is possible that a low number of patients were excluded from the study who were switched in less than 48 h due to rapid deterioration; however, given the low acuity of our patients included in the study, this is unlikely.

The rates of clinical cure found in the current study reflect findings in similar studies conducted with linezolid for the treatment of VRE and doxycycline for the treatment of UTIs. Clinical cure rates using linezolid for the treatment of VRE UTIs vary from approximately 70% to 95%, which strongly correlates with the cure rates found in this retrospective study [16,17]. In the available doxycycline literature, clinical cure rates vary, but a 2023 conference abstract cites UTI cure rates between 75% and 89% amongst a small patient population [21]. The cure rate of 17/18 (94.4%) in the current study was higher than anticipated, indicating a future area of research and an opportunity for oral doxycycline’s use in the treatment of UTIs. Doxycycline’s numerically high cure rate in this trial was unexpected, despite its limited urinary excretion. We hypothesized this is due to this study specifically looking at *Staphylococcus aureus,* where all isolates were susceptible to definitive therapy. This cure rate may differ from historical doxycycline cure rates in UTI trials because this study excluded Gram-negative organisms. These studies included *Pseudomonas aeruginosa*, which is considered tetracycline-resistant [22]. In terms of our study, it is possible the finding reflects chance variation rather than a meaningful underlying association. These findings highlight the potential value of further investigating oral doxycycline as a treatment option for UTIs. As of the publication date of this article, we are not aware of any other dedicated clinical studies published in PubMed that have evaluated the effectiveness of oral antimicrobials for the treatment of MRSA UTIs.

The inclusion of ASB patients for secondary outcomes must also be discussed. The inclusion of these patients represents a more real-world representation of patients with positive urinary cultures treated with antibiotics. Patients without symptoms, despite guideline recommendations to avoid treatment, often do receive treatment, and in the setting of this study, 18/91 (19.8%) of patients were asymptomatic. This may have impacted the change in therapy due to clinical failure and readmission rates in favor of doxycycline and TMP-SMX due to the higher rates of ASB in these groups. The rate of adverse effects did not demonstrate any differences between the groups, as asymptomatic patients would be just as likely to have adverse effects as symptomatic patients. Two ASB patients were included in the primary outcome as not having achieved clinical cure. One patient in the linezolid group was readmitted for reasons attributed to a UTI, and one in the doxycycline group had a change in therapy due to prescriber-perceived lack of improvement. Although ASB was generally excluded from the primary outcome, the two aforementioned patients were included because they affected clinically relevant endpoints. The higher number of symptomatic UTIs in the linezolid group, while not impacting the primary analysis, may have impacted the secondary outcomes. Although not statistically significant, numerically, linezolid patients were readmitted more often than the other two groups, which may be indicative of linezolid patients being more likely to have a true UTI. More notably, the finding of such common treatment of ASB shows a need for further stewardship interventions and education initiatives.

Clinical test of cure and microbial eradication were collected data points, but due to concerns with the availability of results, they were not included as outcomes. However, based on collected information, test of cure within 30 days of treatment was performed in 27 patients in total (22 on linezolid, three on TMP-SMX, and two on doxycycline). Microbial eradication occurred in 19/22 (86.4)% of linezolid patients, 3/3 (100.0%) of TMP-SMX patients, and 1/2 (50.0%) of doxycycline patients. The interpretation of these data points must be made with caution, due to limited sample size. Microbial test of cure for UTIs is also not a common or recommended practice in routine clinical care [23]. If trials are conducted in the future, the inclusion of this objective microbial signal, with appropriate statistical power, would be valuable. If readmission within 30 days of discharge attributable to UTI occurred, repeat culture data were collected, if available. Three patients in the linezolid group and one in the doxycycline group had recurrence of UTI by culture-positive MRSA on readmission. These findings suggest that MRSA was likely not the cause of many readmissions, but instead another non-MRSA organism was the etiology of the UTI. The availability of follow-up data relied on patients following up within the same health system and therefore represents a limitation of this retrospective study. Susceptibility data were limited to the doxycycline and TMP-SMX groups. Linezolid sensitivities were not performed on urinary isolates and therefore were not available upon chart review. No patients were on definitive resistant therapy in the doxycycline or TMP-SMX groups, but this is unknown for the linezolid group. While the lack of linezolid susceptibility is an important consideration, linezolid resistance is exceedingly rare, with a large demographic study conducted over 17 years demonstrating 100% susceptibility in 2697 isolates [24]. Local antibiogram data from the health system in which this study was conducted also demonstrates 100% susceptibility to linezolid in MRSA non-urinary isolates. Treatment selection was provider-directed and therefore represents a limitation of the retrospective nature of the study.

This study includes a high proportion of complicated UTI patients, increasing the applicability to more invasive UTIs. Classification of a complicated UTI was based on the diagnosis of catheter-associated UTI, pyelonephritis, or cystitis with fever in accordance with the 2025 Infectious Diseases Society of America’s definition of cUTI [23]. This is reflected in the common use of IV empiric therapy, which demonstrates that many of the oral therapy agents were used as oral step-down from IV therapy in our study, but this does not represent our entire population. Additionally, the patient population analyzed in the current study is representative of those typically encountered in the clinical setting, as a majority of the study participants had recent healthcare exposure, which aligns with prior literature demonstrating an association between healthcare exposure and the development of MRSA infections [1,2,3]. While there were no observed differences in clinical cure rates between the three antimicrobial groups, significance may be observed in future studies with expanded patient populations. This is especially apparent with the TMP-SMX group, which had eight patients included, with only five included for the primary analysis. While statistical analyses were selected to account for the limited sample size, the small sample remains a potential source of confounding. This was likely due to the elderly patient population represented, with prescribing indicating a desire to avoid the AEs associated with TMP-SMX in this population. The primary outcome of this study was designed to reflect a pragmatic and clinically relevant measure of treatment success.

In further support of the potential of oral antibiotics for the treatment of MRSA UTIs, the rarity of adverse effects related to antimicrobial therapy is evident, with only 2/91 (2.2%) of patients discontinuing therapy. Specifically, thrombocytopenia was reported in four patients on linezolid, gastrointestinal upset occurred in one doxycycline patient, and *Clostridioides difficile* infection occurred in one patient in the TMP-SMX and doxycycline groups. No differences were detected between treatment groups for safety outcomes, indicating well-tolerated drug therapies across all groups.

## 4. Methods

The study described herein is a retrospective cohort study conducted across four acute care centers within a large regional healthcare system in southwest Florida. This study was approved by the healthcare system’s institutional review board (IRB) and conducted in accordance with its guidelines. The study period was between 1 January 2022 and 30 October 2025. Patient charts were accessed retrospectively after IRB approval. All patients with a positive MRSA urinary culture during the study period were evaluated for inclusion in the study. Three study groups were designed to include patients treated with (1) oral linezolid, (2) TMP-SMX, or (3) doxycycline for MRSA UTI.

Patients were included in the study if they were 18 years of age or older with a positive urinary culture for MRSA. Patients must also have received oral antimicrobial therapy with TMP-SMX, linezolid, or doxycycline. Patients were excluded if they had a polymicrobial urinary tract infection, or if they had a concomitant extra-urinary MRSA infection (e.g., bacteremia, pneumonia, or skin and soft tissue infection), as confirmed by culture of a specimen collected from outside of the urinary tract. Patients were also excluded if they received non-study MRSA-active therapy (e.g., antimicrobials other than oral linezolid, doxycycline, or TMP-SMX) for greater than 50% of their antimicrobial therapy course, or if they received less than 48 h of oral linezolid, doxycycline, or TMP-SMX. Patients were excluded from the study if they were diagnosed with prostatitis during the same admission as well. Exclusion criteria were mutually exclusive and applied hierarchically in the order listed above.

The primary outcome was clinical cure of urinary infection, defined as a composite outcome consisting of resolution of baseline urinary tract symptoms without the need for additional antibiotic therapy, no change of therapy due to treatment failure, and no UTI recurrence or hospital re-admission within 30 days of discharge for reasons attributable to the UTI. Patients with ASB were excluded from primary analysis, unless they failed another component of the primary outcome. In these cases, the patient was classified as a negative response, as fulfillment of all three components was no longer possible regardless of the status of symptoms. Secondary outcomes included the components of the primary outcome, as well as the rate of adverse events that led to discontinuation of therapy. Thrombocytopenia was defined as the occurrence of a platelet drop of 50% or greater during the course of therapy.

Continuous, non-normal data were presented as median (25–75% interquartile range) and analyzed using the Kruskal–Wallis test. Dunn’s test post hoc analysis was conducted on statistically significant demographics. Continuous, normally distributed data were presented as means with standard deviations and analyzed using one-way ANOVA. Categorical data were presented using frequencies and percentages and analyzed using the chi-square test, or Fisher’s exact test when the expected sample size was less than 5, with 95% Wilson Score Intervals provided for primary and secondary outcomes. Pairwise comparisons were conducted post hoc on statistically significant demographics.

## 5. Conclusions

In patients with MRSA UTI treated with oral antimicrobials (linezolid, TMP-SMX, and doxycycline), clinical cure ranged from 78.9% to 94.4%, with no observed differences in clinical cure between oral antimicrobial treatment groups. Similarly, no differences were observed between the secondary outcomes of readmission due to UTI, symptom resolution, change in therapy due to failure, or discontinuation of therapy due to AEs between treatment groups. These findings suggest that the three oral treatment strategies may be associated with similar clinical outcomes and could be considered for the treatment of MRSA UTIs. Larger, adequately powered studies across multiple healthcare systems are needed to confirm these findings and further define optimal oral treatment strategies for MRSA UTI.

## Figures and Tables

**Figure 1:**
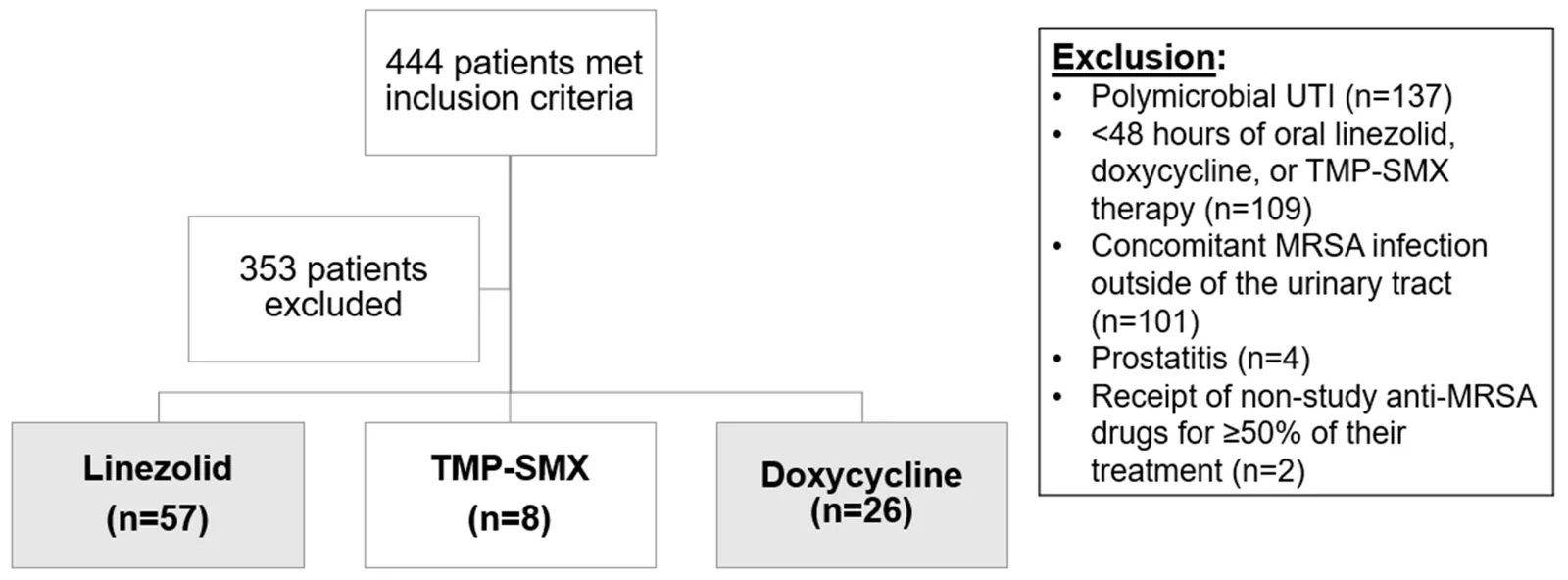
**Study cohort flowchart.** Description of patient selection and exclusion criteria. UTI, urinary tract infection; TMP-SMX, trimethoprim-sulfamethoxazole; MRSA, methicillin-resistant *Staphylococcus aureus.*

**Figure 2:**
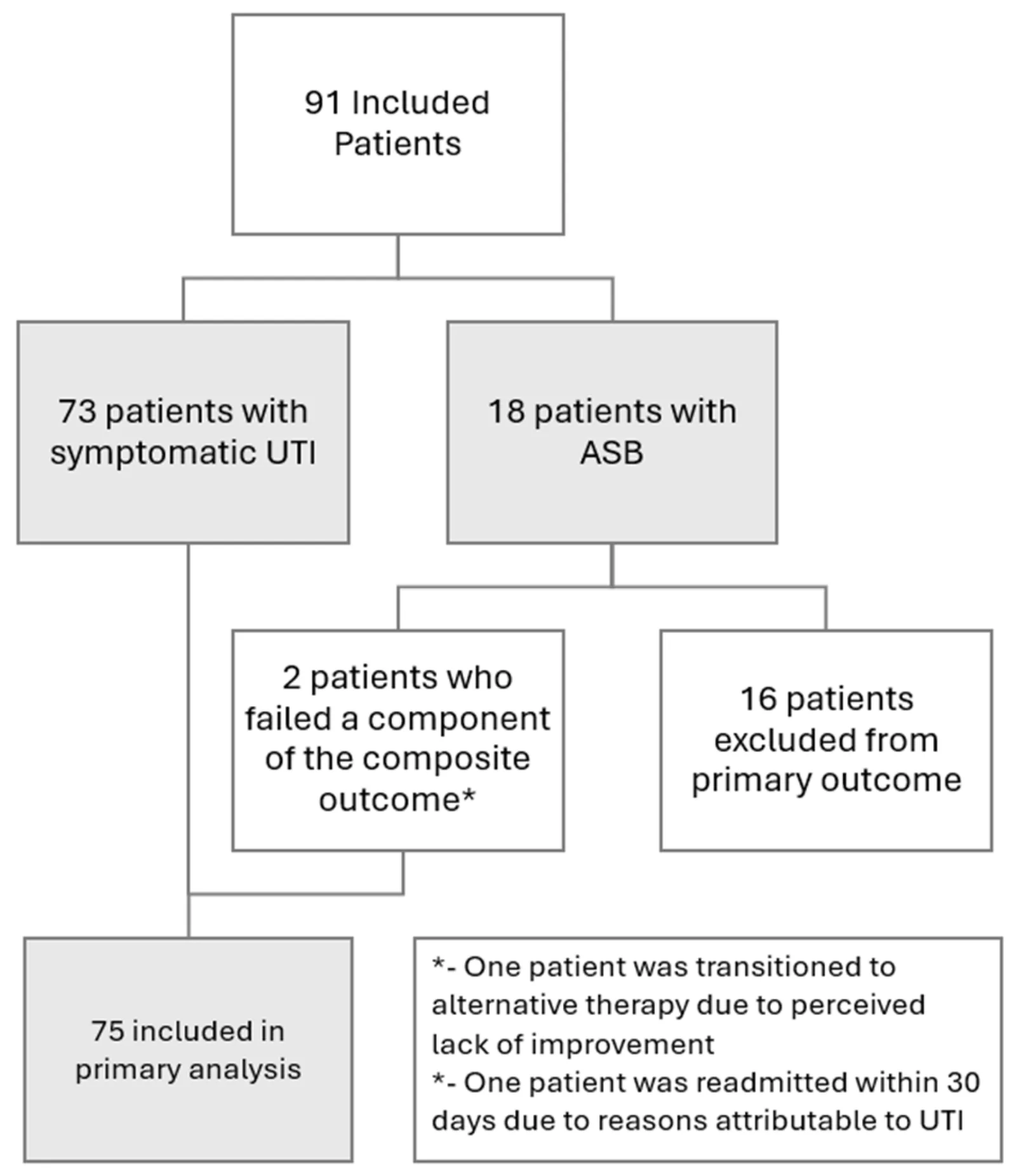
**Primary outcome patient inclusion.** Description of patient selection for inclusion in primary outcome; ASB, asymptomatic bacteriuria; UTI, urinary tract infection.

**Table 1:** Patient Demographics (*n* = 91).

Treatment Group	Linezolid	TMP-SMX	Doxycycline	*p*-Value
Cohort size (*n*)	57	8	26	-
Age (years), mean ± SD	77.3 ± 13.1	75.3 ±10.2	82.6 ± 9	0.12
Male sex	42 (73.7)	5 (62.5)	16 (61.5)	0.49
Weight (kilogram), mean ± SD	77.6 ± 20.1	74.6 ± 22	75.9 ± 31.8	0.93
Creatinine clearance < 30 mL/min	17 (29.8)	0 (0)	5 (19.2)	0.14
Chronic kidney disease	22 (38.6)	1 (12.5)	5 (19.2)	0.10
Dialysis	4 (7)	0 (0)	1 (3.9)	0.65
Immunosuppressed	9 (15.8)	0 (0)	0 (0)	0.07
ICU admission	3 (5.3)	1 (12.5)	4 (15.4)	0.30
Diabetes mellitus (type 1 or 2)	24 (42.1)	2 (25)	10 (38.6)	0.64
Hypertension	38 (66.7)	4 (50)	16 (61.5)	0.63
Recurrent UTI	36 (63.2)	7 (87.5)	14 (53.9)	0.23
Recent hospitalization within 90 days	42 (73.7)	6 (75)	16 (61.5)	0.50
Recent IV antibiotic use within 90 days	38 (66.7)	5 (62.5)	14 (53.8)	0.53
Prior isolation of MRSA in urine within 1 year	15 (26.3)	2 (25)	7 (26.9)	0.99
Urinary catheter present prior to admission	11 (19.3)	0 (0)	6 (23.1)	0.33
Complicated UTI	31 (54.4)	2 (25)	12 (46.15)	0.28
Length of stay (days), median [IQR]	7.2 [4.9–12.9]	8.6 [4.1–10.8]	8.1 [5–12.9]	0.86
Duration of MRSA therapy (days), median [IQR]	10 [7.3–13]	6 [5.5–9.2]	9.4 [6.3–11.4]	0.03
Empiric therapy active against MRSA	33 (57.9)	4 (50)	18 (69.2)	0.51
Oral empiric therapy	12 (21.1)	3 (37.5)	6 (23.1)	0.59
Asymptomatic bacteriuria	6 (10.5)	3 (37.5)	9 (34.6)	0.02
Day of therapy when oral transition occurred (days), median [IQR]	1.6 [0.8–2.8]	1.3 [0–2.3]	1.5 [0.2–2.5]	0.43
Dose of antimicrobial appropriate for renal function	-	8 (100)	-	-

Data are represented as *n* (%) unless otherwise specified; IQR: interquartile range; SD: standard deviation; mL/min: milliliters per minute; UTI: urinary tract infection.

**Table 2:** Outcomes (*n* = 91).

Primary Outcome	Linezolid	TMP-SMX	Doxycycline	*p*-Value
Clinical cure (*n* = 75 ^)	41/52 (78.8, 66.0–87.8)	4/5 (80, 37.6–96.4)	17/18 (94.4, 74.2–99.0)	0.24
Secondary outcomes	Linezolid	TMP-SMX	Doxycycline	*p*-value
Resolution of baseline UTI symptoms (*n* = 73 *)	48/51 (94.1, 84.1–98.0)	4/5 (80, 37.6–96.4)	16/17 (94.1, 73.0–99.0)	0.37
Change in therapy due to treatment failure (*n* = 91)	1/57 (1.9, 0.3–9.3)	0/8 (0, 0.0–32.4)	1/26 (3.8, 0.7–18.9)	0.61
Hospital readmission within 30 days of discharge attributable to UTI (*n* = 91)	9/57 (15.8, 8.5–27.4)	0/8 (0, 0.0–32.4)	0/26 (0, 0.0–12.9)	0.07
Adverse effects that led to discontinuation of therapy (*n* = 91)	2/57 (3.5, 1.0–11.9)	0/8 (0, 0.0–32.4)	0/26 (0, 0.0–12.9)	1.00

Data represented as *n* (%, 95% CI) unless otherwise specified; UTI, urinary tract infection; ^ See Figure 2 for primary outcome patient inclusion; * Patients not included in secondary outcome due to lack of baseline urinary symptoms.

## Data Availability

The data presented in this study are available on request from the corresponding author due to privacy concerns and IRB requirements.
